# Development and validation of a questionnaire for laboratory medicine knowledge and attitudes in clinical medical interns

**DOI:** 10.3389/fmed.2025.1726666

**Published:** 2025-12-16

**Authors:** Yonggang Yang, Shuihua Xu, Song Chen, Jiyun Tian, Baobing Chen

**Affiliations:** 1 Department of Clinical Laboratory, Hangzhou Third People’s Hospital, Hangzhou, Zhejiang, China; 2 Department of Dentistry, Tianshui Wulin Subdistrict Community Health Service Center, Gongshu District, Hangzhou, Zhejiang, China

**Keywords:** clinical medical intern, questionnaire validation, laboratory medicine knowledge, undergraduate medical education, attitudes

## Abstract

**Introduction:**

In medical education systems, the clinical internship phase is critical for clinical laboratory knowledge training. Insufficient clinical laboratory knowledge among clinicians directly leads to diagnostic errors or suboptimal treatment decisions. This study aimed to develop and validate a questionnaire evaluating laboratory medicine knowledge and attitudes among clinical medical undergraduates during their internships.

**Methods:**

Based on a comprehensive literature review and focus group discussions, our research team designed the “Questionnaire on Laboratory Medicine Knowledge and Attitudes Among Undergraduate Clinical Medical Interns.” The questionnaire comprises four sections: demographic information, knowledge assessment, attitudes evaluation, and suggestions for improvement. Internal consistency was analyzed using Cronbach’s α and intraclass correlation coefficients (ICC). Content validity was assessed via Content Validity Index (CVI) and Content Validity Ratio (CVR). Structural validity was examined using exploratory factor analysis (EFA), confirmatory factor analysis (CFA), and model fit indices (e.g., GFI, CFI, RMSEA).

**Results:**

A total of 303 valid questionnaires were collected from interns across 11 general hospitals in the Yangtze River Delta region. The Cronbach’s α coefficients were 0.905 (knowledge subscale) and 0.803 (attitudes subscale), respectively, with ICC values of 0.705 and 0.576. The CVR was 0.923, and all item-level CVIs exceeded 0.78. EFA revealed a two-factor structure (KMO = 0.783, Bartlett’s test *p* < 0.001), accounting for 79.357% of the total variance. CFA confirmed satisfactory model fit (χ^2^/df = 2.214, RMSEA = 0.063, GFI = 0.988, NFI = 0.992, CFI = 0.995, TLI = 0.984).

**Conclusion:**

This validated questionnaire demonstrates preliminary reliability and validity for assessing laboratory medicine knowledge and attitudes among clinical interns in specific healthcare contexts (e.g., urban tertiary hospitals). Further validation in diverse healthcare contexts is required before broader implementation.

## 1 Introduction

Clinical laboratory medicine serves as a cornerstone in disease diagnosis and treatment. However, in the current medical education system, clinical medical students receive few or no systematic rotations in clinical laboratories, resulting in insufficient laboratory medicine knowledge (1, 2). This training gap is a common concern internationally. For instance, a 2014 status report on U.S. medical schools indicated that while most (84%) offered laboratory medicine coursework, the vast majority placed it in the pre-clinical years, with only a modest proportion (19%) providing such training during the clinical clerkship years (2). Similarly, a recent survey of interns in Ireland regarding dermatology, a discipline reliant on laboratory diagnostics, suggested limited exposure to specialized training during clinical rotations, hinting at a potential shortfall in practical laboratory medicine experience (3). As pre-clinical curricula often focus on theoretical knowledge without sufficient integration into clinical decision-making contexts, medical interns frequently enter their clinical practice with inadequate preparation in laboratory medicine.

Graduates are expected to meet the basic requirement of selecting appropriate clinical examination methods based on patients’ conditions, safety considerations, and cost-effectiveness, while also demonstrating the ability to interpret and explain test results (1). These expected competencies are explicitly outlined in educational objectives, such as the exit competencies for graduating medical students established by Canadian scholars, which include core skills like test selection and result interpretation (4). Consequently, the clinical internship phase, which bridges theory and practice, provides a critical opportunity for students to engage with how laboratory medicine informs clinical decision-making in real-world settings. Their ability to appropriately select tests [avoiding diagnostic delays and unnecessary costs associated with inappropriate test utilization (5)], accurately interpret reports [reducing diagnostic errors (5, 6)], and understand the impact of test results on diagnostic accuracy (6), directly reflects their application of clinical reasoning and medical knowledge. This further underscores the growing importance of clinical reasoning and medical knowledge in modern healthcare practice.

Since Gottfried et al. (7), and Smith et al. (8, 9) emphasized the importance of laboratory medicine education, international attention to laboratory medicine curricula has grown. In France, Reix et al. (10) developed a laboratory knowledge satisfaction questionnaire based on clinical laboratory training manuals to assess training effectiveness. Ford et al. (4) in Canada established competency standards for medical graduates in laboratory medicine. An Iranian study (11) from Shiraz University of Medical Sciences surveyed medical students’ satisfaction with pathology courses, but focused solely on *post hoc* evaluation. Saffar et al. (12) later designed a knowledge assessment tool for undergraduates in laboratory medicine, though with only 37 participants and a non-scale format. Smith’s team at Yale University (13) developed a 10-item microbiology questionnaire aligned with US medical licensing exams. In Tunisia, Bengayed and Hafien (14) created a transfusion medicine knowledge tool. However, laboratory medicine encompasses broader disciplines including biology, immunology, chemistry, hematology, molecular biology, and genetics (15).

Existing tools lack comprehensive assessments of both laboratory medicine knowledge and attitudes among clinical medical interns. Clinical internships provide a critical opportunity for students to engage in real-world clinical settings, bridging the gap between theoretical knowledge and practical application. During this phase, students witness firsthand how laboratory medicine supports clinical decision-making in authentic healthcare settings. This study developed and validated a questionnaire to assess laboratory medicine knowledge and attitudes among undergraduate clinical medical interns, evaluating its reliability and validity.

## 2 Materials and methods

### 2.1 Study design and setting

This cross-sectional study was conducted from March 2025 to April 2025 in 11 general hospitals across the Yangtze River Delta region of China. Participating sites included tertiary care institutions with active clinical internship programs for undergraduate medical students.

### 2.2 Instrument development and validation: the CLKAQ

#### 2.2.1 Conceptual framework and item generation

The development of the Clinical Laboratory Knowledge and Attitudes Questionnaire (CLKAQ) employed a mixed-methods survey design, an approach widely recognized in Health Professions’ Education (HPE) (3) for its robustness. The questionnaire’s conceptual foundation was guided by established competency frameworks, including the *ACGME Common Program Requirements (Residency)*(16) from the Accreditation Council for Graduate Medical Education (ACGME), the *Chinese Undergraduate Medical Education Standards for Clinical Medicine (2022 Edition) General Objectives Requirement* (1), and a synthesis of existing literature. The instrument’s domains were explicitly delineated according to two primary competency standards. The Knowledge domain was conceptualized to align with the ACGME requirement for “Practice-based Learning and Improvement” and the Chinese standards, both emphasizing the ability to select appropriate diagnostic tests and accurately interpret results for clinical decision-making. Concurrently, the Attitudes domain was developed based on contemporary medical education literature, focusing on fostering interdisciplinary collaboration, recognizing the value of laboratory medicine in patient care, and promoting motivations for lifelong learning.

To ensure a comprehensive foundation for item development, a systematic scoping review was conducted. The review methodology involved queries across five electronic databases—PubMed, China National Knowledge Infrastructure (CNKI), Chinese Medical Association Full-text Journal Database, Springer LINK, and Web of Science—for publications from January 2015 to December 2024. The inclusion criteria targeted studies involving undergraduate clinical medical students during or after their clinical internship, with a core focus on educational interventions, teaching methods, or curricula designed to integrate laboratory medicine knowledge into clinical training. This encompassed dedicated laboratory sessions, case-based courses on test selection and interpretation, simulation-based learning, and other interventions aimed at enhancing interpretive skills. A wide range of study types, including original research, curriculum evaluations, and reviews, published in English or Chinese and accessible in full text, were included. This scoping review facilitated a mapping of the educational landscape and informed the initial item pool by integrating diverse pedagogical insights and contemporary challenges.

The resultant CLKAQ, designed for clinical medical undergraduates during their internships, was grounded in a rigorous synthesis of international competency standards and informed by literature on questionnaire development methodologies in medical education (12, 17, 18) to guide item generation and scale construction, with further refinement through insights from focus group discussions and predictive analyses.

#### 2.2.2 Questionnaire structure

The CLKAQ comprised four sections: (1) Basic Information (demographics), (2) Knowledge (four 5-point Likert-scale items), (3) Attitudes (three 5-point Likert-scale items and four multiple-choice questions), (4) Suggestions (open-ended questions on institutional educational improvements with binary yes/no responses and qualitative recommendations for training optimization), free-text responses were manually reviewed to eliminate accidental identifiers (Table 1; Supplementary Material 1). The expert panel consisted of 10 multidisciplinary professionals: Two medical educators, two hospital internship administrators, four clinicians, and two laboratory medicine specialists. Prior to the expert panel review, a focus group discussion was conducted with a separate cohort of 10 clinical interns (who did not participate in the main survey) to gather feedback on the clarity, relevance, and comprehensibility of the initial questionnaire items. Their insights were used to refine the item wording and structure.

**TABLE 1:** CLKAQ among undergraduate clinical medical interns.

Structure	Item	Content
Part I (Basic information)		Gender, age, city
Part II (Knowledge)	Q1-Q4	“I believe my clinical laboratory medicine knowledge (e.g., report interpretation, correlation between test results and clinical diagnosis) meets the job requirements of my current institution.”
	Q1	
	Q2	“I am fully aware of the clinical laboratory medicine knowledge required for my target position.”
	Q3	“I can independently perform preliminary interpretation of common laboratory reports (e.g., complete blood count, biochemistry, coagulation function).”
	Q4	“I understand and can implement the critical value reporting system in clinical laboratory medicine.”
Part III (Attitudes)	Q5-Q11	“I consider clinical laboratory medicine knowledge crucial for clinical decision-making.”
	Q5	
	Q6	“Acquiring more laboratory medicine knowledge provides a competitive advantage in job-seeking.”
	Q7	“I hope to gain more clinical laboratory medicine knowledge.”
	Q8	“What types of clinical laboratory medicine knowledge do you most want to learn?” *(Multiple-choice options)*
	Q9	“What are your primary motivations for learning clinical laboratory medicine?” *(Multiple-choice options)*
	Q10	“How do you typically access clinical laboratory medicine knowledge?” *(Multiple-choice options)*
	11	“What do you perceive as the main barriers to learning clinical laboratory medicine?” *(Multiple-choice options)*
Part IV (Suggestions)	Q12-Q13	“Do you think hospitals or medical schools should increase education on clinical laboratory medicine?” *(Yes/No)*
	Q12	
	Q13	“What suggestions do you have for improving clinical laboratory medicine training in hospitals or medical schools?” *(Open-ended; e.g., “Monthly online training,” “Quarterly case discussions”)*

#### 2.2.3 Participants and data collection

Sample size determination followed psychometric standards recommending 10–30 participants per scale item (19). For the 7 core Likert-scale items, this yielded a minimum target of 210 participants. This cross-sectional study was conducted in 11 general hospitals across China’s Yangtze River Delta region. Questionnaires were distributed by hospital administrators via WeChat groups to undergraduate clinical medical interns within these institutions, achieving response rates ranging from 42 to 63% across different hospitals. Prior to initiating the study, informed consent was obtained from all participants. Confidentiality and anonymity of the collected data were strictly guaranteed throughout the research process.

Inclusion Criteria: (1) Age ≥ 20 years; (2) Voluntary participation; (3) Active undergraduate clinical medicine interns currently undertaking hospital rotations. Exclusion Criteria: Responses exhibiting logical inconsistencies, excessively short completion times (< 2 min), extreme response patterns, invalid answers to open-ended questions, or duplicate submissions.

The electronic data collection was meticulously carried out utilizing the WeChat-based “Questionnaire Star” mini-program. The questionnaire was digitally constructed and configured within the platform, and a unique QR code was generated and disseminated simultaneously to all potential participants via WeChat groups managed by hospital administrators. Participants accessed the survey by scanning the QR code. Following data retrieval, a rigorous two-phase screening process was implemented: automated checks within the platform filtered out incomplete submissions, followed by manual screening based on predefined exclusion criteria.

#### 2.2.4 Validation

##### 2.2.4.1 Validity and reliability assessment

This study primarily utilized SPSS 25.0 and AMOS 26.0 for statistical analyses. Content validity was assessed via Content Validity Ratio (CVR) for individual items and Content Validity Index (CVI) for questionnaire sections, with items retained only if overall CVR > 0.741 and *p* < 0.05 (20). Formulae for CVI calculation were applied as follows: number of experts rating the item as relevant divided by total number of experts, while CVR was calculated using (Ne – N/2)/(N/2), where Ne represents experts deeming the item relevant and necessary.

The internal consistency reliability assessment, along with the exploratory and confirmatory factor analyses (EFA/CFA), were conducted exclusively on the seven core Likert-scale items (Q1–Q7) designed to measure the latent constructs of “Knowledge” and “Attitudes.” The multiple-choice items (Q8–Q11) and open-ended questions (Q12–Q13) were not included in these psychometric analyses, as they serve different measurement purposes: the multiple-choice items aim to capture specific learning preferences, motivations, channels, and barriers, while the open-ended questions collect qualitative suggestions. The validity of these multiple-choice items was established through rigorous content validity assessment by the expert panel and face validity testing via the pre-study focus group discussion. Internal consistency was evaluated using Cronbach’s α and the Intraclass Correlation Coefficient (ICC) (21); Structural validity was examined through EFA employing principal component analysis and varimax rotation (22), supported by Kaiser-Meyer-Olkin (KMO) and Bartlett’s Test of Sphericity (23). CFA validated construct validity with maximum likelihood estimation, and model fit was appraised using Hu and Bentler (24) criteria: chi-square/df ratio (χ^2^/df) < 5 (preferably < 2), root mean square error of approximation (RMSEA) < 0.08, and goodness-of-fit index (GFI), normed fit index (NFI),comparative fit index (CFI), and Tucker-Lewis index (TLI) > 0.90. All indices are detailed in Table 2.

**TABLE 2:** Fit indices value ranges and ideal standards.

Fit index	Value range	Ideal standard
χ^2^/df	> 0	< 5, Preferably <2
RMSEA	> 0	< 0.08 (Indicating good fit)
GFI	0–1	> 0.9 (Indicating good fit)
NFI	0–1	> 0.9 (Indicating good fit)
CFI	0–1	> 0.9 (Indicating good fit)
TLI	0–1	> 0.9 (Indicating good fit)

##### 2.2.4.2 Qualitative content analysis

The qualitative analysis focused on the text data provided in response to the open-ended question Q13. All participants who completed the survey were eligible to contribute responses, which were collected electronically via a free-text field without facilitated discussion. The data were analyzed using a directed content analysis approach (25). The coding framework was developed iteratively by two doctoral researchers. Its initial version was informed by the study’s theoretical foundation (e.g., ACGME competencies) and insights from pre-study focus groups. The framework was refined through pilot coding of a subset of responses and discussions until a consensus on code definitions and application rules was achieved. The two researchers then independently coded the entire dataset using the finalized framework. To ensure coding reliability, inter-rater agreement was formally assessed. Discrepancies were addressed through a structured process: primarily via consensus discussions between the coders, with unresolved cases arbitrated by the principal investigator. The textual data were transcribed and managed by the research team during the analysis. Methodological trustworthiness was upheld through several strategies: (1) triangulation of qualitative themes with quantitative results; (2) maintenance of a detailed audit trail documenting all coding decisions; and (3) regular peer debriefing sessions within the research team to review interpretations. The analytical outcomes included the quantification of thematic frequencies and the selection of representative quotations to illustrate the findings.

### 2.3 Ethical considerations

This study involved the development of a novel questionnaire. Our study was approved by the Ethics Committee of Hangzhou Third People’s Hospital (Approval Number: 2025KA064). This study was conducted in compliance with China’s Ethical Review Measures for Biomedical Research Involving Humans and the Declaration of Helsinki. Consent for publication was waived as the anonymized dataset contains no personally identifiable information (in accordance with ICMJE Recommendation 4.2), participants were informed that completing the questionnaire would serve as implied consent.

## 3 Results

### 3.1 Response rates and sample characteristics

A total of 324 questionnaires were collected through convenience sampling. After applying the predefined exclusion criteria, 21 responses were removed, resulting in a final analytical sample of 303 participants. The overall response rate varied across the participating hospitals, ranging from 42 to 63%. All 303 participants responded to the open-ended question (Q13), resulting in a 100% response rate for the qualitative component. This final sample size substantially exceeded the psychometric target, thereby providing robust statistical power for the factor analysis and minimizing the risks of model overfitting and factor instability. The demographic characteristics of the participants are summarized in Table 3.

**TABLE 3:** Sociodemographic characteristics of the participant interns (*N* = 303).

Characteristic	Category	*n*	(%)
Age (years)	20–22 years	75	24.8
	23–25 years	228	75.2
Gender	Male	135	44.6
	Female	168	55.4
City tier	Tier-1 cities	137	45.2
	Tier-2 cities	84	27.7
	Tier-3 cities	82	27.1

### 3.2 Reliability and internal consistency

Statistical analysis revealed the following reliability and validity metrics for the knowledge and attitudes subscales: The Cronbach’s α coefficients were 0.905 (knowledge subscale) and 0.803 (attitudes subscale), respectively [both exceeding 0.7, indicating acceptable reliability (26)]; ICC were 0.705 (knowledge) and 0.576 (attitudes), with values < 0.5, 0.5–0.75, 0.75–0.9, and > 0.90 classified as poor, moderate, good, and excellent reliability, respectively (21). The ICC values indicated moderate-to-good reliability for the Knowledge subscale and moderate reliability for the Attitudes subscale (Table 4).

**TABLE 4:** Reliability, and EFA of the CLKAQ scales (*N* = 303).

Dimension n	Reliability		EFA	
	Cronbach’s α	ICC	Factor 1 (Knowledge)	Factor 2 (Attitudes)
Knowledge	0.905	0.705	0.841	
			0.879	
			0.885	
			0.839	
Attitudes	0.803	0.576		0.752
				0.848
				0.868

Reliability and Exploratory Factor Analysis EFA were performed on the two dimensions (Knowledge and Attitudes) comprising the seven Likert-scale items. The multiple-choice items were excluded from this analysis. CLKAQ, Clinical Laboratory Knowledge and Attitudes Questionnaire; ICC, Intraclass Correlation Coefficient; EFA, Exploratory Factor Analysis; Factor loadings < 0.40 are omitted for clarity. Cales were scored on a 5-point Likert scale (1 = Strongly Disagree, 5 = Strongly Agree); Overall model fit: Extraction Method: Principal Component Analysis, Rotation Method: Varimax, Cumulative Variance Explained: 79.357%, KMO measure: 0.783, Bartlett’s Test of Sphericity: χ^2^ = 1574.626, *p* < 0.001.

### 3.3 Validity evidence

All items exhibited individual Content Validity Index (I-CVI) values > 0.8, and the overall CVR reached 0.923 (Supplementary Material 2). Building on this content validity foundation, EFA was conducted to identify latent factors and assess structural validity. Results showed a KMO measure of 0.783, Bartlett’s test of sphericity yielded statistical significance (*p* < 0.001), and the analysis extracted two factors accounting for 79.357% of the total variance, though such a high proportion may reflect overlapping constructs or item redundancy (Table 4). Factor loadings exceeded 0.5 for all items, confirming robust structural validity.

To further validate the internal structure of the knowledge subscale, CFA was conducted using AMOS 26.0 on the CLKAQ data, building upon EFA. Six validation criteria were analyzed: χ^2^/df, RMSEA, GFI, NFI, CFI, and TLI. Results (Table 5) demonstrated a stable two-factor convergent validity, with all key indices meeting established criteria.

**TABLE 5:** CFA model fit indices for the CLKAQ.

Model fit index	χ ^2/^df	RMSEA	GFI	NFI	CFI	TLI
Two-factor model	2.214	0.063	0.988	0.992	0.995	0.984
Threshold for acceptance	<3	<0.06	>0.90	>0.90	>0.95	>0.95
Interpretation	Excellent	Good	Good	Good	Excellent	Excellent

A confirmatory factor analysis (CFA) was performed to test the two-factor model (Knowledge and Attitudes) comprising the seven Likert-scale items. Model fit was evaluated using multiple indices. The root mean square error of approximation (RMSEA) value of 0.063 indicates a reasonable fit according to the criteria proposed by MacCallum et al. (27), who suggest that values up to 0.08 represent a reasonable error of approximation in the population. Furthermore, the comparative fit index (CFI) and Tucker-Lewis index (TLI) both exceeded the recommended threshold of 0.95, and the chi-square/degrees of freedom ratio (χ^2^/df) was below 3. The collective pattern of these fit indices supports the acceptability of the two-factor model (28).

**FIGURE 1:**
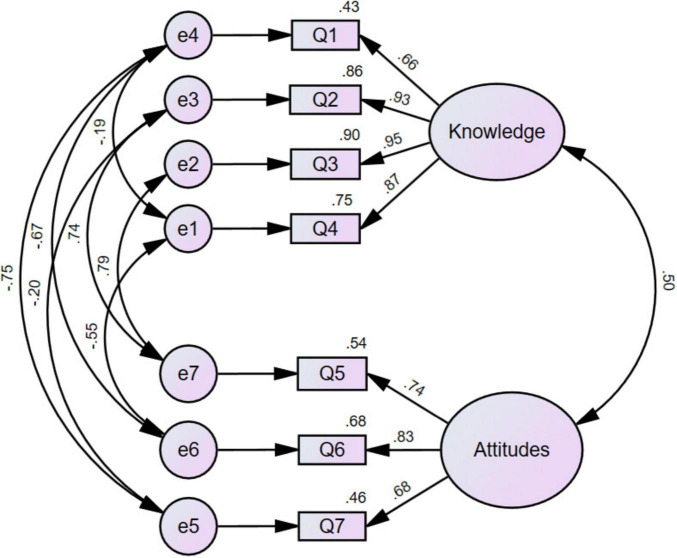
Revised path diagram of CFA for the CLKAQ.

This study established a two-factor structural model. However, the initial two-factor model exhibited suboptimal fit indices (χ^2^/df = 18.644, RMSEA = 0.242, CFI = 0.854), and circular causal relationships were identified among items. Consequently, based on modification indices and theoretical justification, error covariance paths were added, this adjustment improved model fit without compromising the conceptual integrity of the factors. The final model not only satisfied the criteria, but also demonstrated superior alignment with the theoretical framework of “medical laboratory knowledge and attitudes,” The revised confirmatory factor analysis path diagram for the medical laboratory knowledge and attitudes scale is presented in Figure 1. The revised CFA model (Figure 1) showed improved fit indices, confirming the hypothesized two-factor structure.

### 3.4 Results of qualitative analysis

Qualitative analysis of the 303 open-ended responses to Q13 provided rich, detailed insights into the interns’ proposed strategies for improving laboratory medicine training. Directed content analysis of the textual data generated 65 initial codes, which were iteratively synthesized into five overarching themes, as detailed in Table 6. These themes encompass a spectrum of practical and formalized training approaches, reflecting a strong intern preference for structured and diverse educational formats. The most prevalent recommendation, endorsed by 83 interns (27.4%), was the implementation of Mandatory Clinical Laboratory Rotations. This theme highlighted a pressing need to gain operational understanding and practical knowledge within the laboratory department, as exemplified by the suggestion: “A compulsory rotation of at least 2 weeks in the lab department is essential to understand the entire process and quality control.” The complete distribution of themes, along with their prevalence and representative quotations, is presented in Table 6. Collectively, these findings provide a clear and actionable framework for refining laboratory medicine curricula during clinical clerkships, directly addressing the perceived gaps in current educational practices.

**TABLE 6:** Thematic analysis of suggestions for improving laboratory medicine training (*N* = 303).

Theme	n	Prevalence n (%)	Representative quote(s)
1. Mandatory clinical laboratory rotations	83	27.4%	“A compulsory rotation of at least 2 weeks in the lab department is essential to understand the entire process and quality control.” “We need hands-on experience in specialized fields like molecular biology to see how it’s done in real practice.”
2.Test report interpretation workshops	64	21.1%	“Regular workshops led by lab physicians would help us bridge the gap between theory and practice in interpreting reports.” “Quarterly case-based sessions on how to read CBC, coagulation, and biochemistry panels would be very beneficial.”
3. Hospital academic lectures on emerging technologies	63	20.1%	“Invite experts to give lectures on new technologies like molecular diagnostics and their clinical applications.” “We need to learn about the principles and limitations of new lab techniques through quarterly technical seminars.”
4. Monthly microlearning online modules	60	19.8%	“Short, focused online modules (< 1 h) each month on specific tests would be easier to fit into our busy schedule.” “Bite-sized learning content that we can access on our phones would be very efficient.”
5. Case-based diagnostic sessions	33	10.9%	“Learning through real, anonymized patient cases where we have to select and interpret tests would develop our clinical reasoning.” “Simulated diagnostic scenarios using real patient data would make the learning stick.”

n = number of interns whose responses were coded into this theme; % = percentage of the total sample (*N* = 303). The sum of n exceeds 303 and percentages exceed 100% as responses could be coded into multiple themes.

### 3.5 Subgroup analyses

To explore potential heterogeneity in responses, subgroup analyses were conducted based on key demographic variables (gender, age groups, and hospital city-tier) for both knowledge and attitude scale scores. Non-parametric tests (Mann-Whitney U and Kruskal-Wallis H tests) revealed several statistically significant differences (*p* < 0.05). For instance, interns from tier-3 cities demonstrated consistently higher self-perceived competence across multiple dimensions compared to their counterparts in tier-1/2 cities. However, as the primary aim of this paper is to report the development and validation process of the CLKAQ instrument itself, a detailed exposition of these subgroup comparisons falls outside its scope. A comprehensive analysis and discussion of the factors influencing knowledge and attitudes, including the implications of these subgroup differences, are provided in our separate, in-depth research article based on the same dataset (29) (Supplementary Material 3).

## 4 Discussion

### 4.1 Principal findings and instrument overview

This study developed and validated the Clinical Laboratory Knowledge and Attitudes Questionnaire (CLKAQ), a novel instrument designed to address a recognized gap in the assessment of laboratory medicine competencies during clinical internships (2, 5). The quantitative results establish the CLKAQ as a robust tool. The knowledge subscale exhibited excellent internal consistency (Cronbach’s α = 0.905), while the attitudes subscale demonstrated acceptable reliability (Cronbach’s α = 0.803) (26, 30). The high Content Validity Ratio (CVR = 0.923) and favorable item-level Content Validity Indices (I-CVI > 0.78) confirm that the items are relevant and representative of the construct, as judged by experts (20, 31). Structurally, exploratory factor analysis (EFA) extracted a clear two-factor solution (“Knowledge” and “Attitudes”) which was subsequently validated by confirmatory factor analysis (CFA). The CFA model fit indices (χ^2^/df = 2.214, RMSEA = 0.063, CFI = 0.995) all met or exceeded conventional thresholds for good model fit (24, 28), confirming the hypothesized structure. The high cumulative variance explained (79.357%) by the two factors in EFA indicates a cohesive and well-defined measurement model.

### 4.2 Comparison with existing literature and instrument advantages

The CLKAQ advances the field by integrating elements that previous tools have addressed only in isolation. Earlier instruments often focused on specific aspects, such as *post hoc* satisfaction with pathology courses (11), competency standards for graduates (4), or knowledge within a narrow specialty like transfusion medicine (14). In contrast, the CLKAQ provides a concurrent assessment of both knowledge and attitudes, tailored specifically to the clinical internship phase where theoretical knowledge is applied in practice. This integrative approach is crucial, as attitudes toward interdisciplinary collaboration directly influence how laboratory knowledge is utilized in clinical decision-making (32). Furthermore, while tools like the one developed by Saffar et al. (12) assessed knowledge alone with a limited sample, the CLKAQ, grounded in international competency frameworks like the ACGME requirements (16) and Chinese national standards (1), offers a more scalable and psychometrically robust alternative. The inclusion of qualitative feedback (Part IV of the questionnaire) aligns with the approach of Reix et al. (10) but expands its application from evaluating a specific training manual to generating broad, actionable suggestions for curriculum enhancement, thereby providing a more comprehensive evaluation mechanism.

### 4.3 Methodological considerations and psychometric properties

The development process involved strategic methodological choices to ensure both scientific rigor and practical utility (33). The decision to maintain a concise questionnaire with two primary scales was informed by evidence on survey engagement, which suggests that excessive length can negatively impact participation and data quality in web-based surveys (34, 35). The psychometric properties, while strong, also highlight areas for methodological reflection (36). The high variance explained in EFA, though indicative of a strong common construct, could also suggest a degree of item redundancy or a potential limitation in capturing the full breadth of the “attitudes” domain, which may account for its lower ICC value (0.576) compared to the knowledge subscale (21). The initial suboptimal fit in the CFA and the subsequent need for model modification based on modification indices are not uncommon in scale development (37). The final model’s excellent fit confirms that the adjustments were statistically and theoretically justified, resulting in a stable and valid two-factor structure that is psychometrically sound for use in similar educational contexts (24, 28).

### 4.4 Educational implications and future directions

The findings from this study, particularly the rich qualitative data, provide a clear and evidence-based roadmap for reforming laboratory medicine education. The strong intern preference for “Mandatory Clinical Laboratory Rotations” (27.4%) and “Test Report Interpretation Workshops” (21.1%) directly addresses the well-documented theory-practice gap in medical training (2, 5, 12). These suggestions call for experiential learning opportunities that move beyond the passive knowledge acquisition typical of pre-clinical years. To effectively implement these changes, educational strategies such as Interprofessional Education (IPE) can be highly valuable. As Weber and Mirza (32) argued, integrating medical students with laboratory professionals through IPE fosters mutual understanding and improves future collaboration. Additionally, innovative credentialing models like the digital microcertification implemented by Graham et al. (38) for transfusion medicine could be adapted to motivate interns and formally recognize their competency in key areas such as test interpretation or critical value reporting. These strategies, derived directly from learner feedback, represent feasible and forward-looking approaches to cultivating physicians who are better prepared to utilize laboratory medicine effectively in patient care.

## 5 Limitations and future directions

This study has several limitations. First, the cross-sectional design precludes causal inferences. Second, the regional specificity of the sample (Yangtze River Delta) may limit the generalizability of the findings to other cultural or institutional contexts. Third, unmeasured variables such as internship duration and specific departmental rotations may introduce heterogeneity; future studies should employ stratified analyses to isolate these effects. Furthermore, this study relied on self-reported measures and did not incorporate external assessments, such as evaluations from supervising physicians. Therefore, it remains unclear whether interns with high self-perceived scores are also rated as more competent in clinical practice. Similar to challenges in validating other self-report scales (39), the attitude measurements may be susceptible to social desirability bias. Future iterations could incorporate social desirability scales to control for this effect. A key methodological consideration is that the “knowledge” construct was assessed via self-report, capturing perceived confidence rather than objective mastery (40, 41). However, the utility of the CLKAQ lies in its role as a catalyst for self-reflection and self-regulated learning (42–44). Finally, the two-factor structure may oversimplify the complexity of laboratory medicine competencies. Future research should combine self-assessment with supervisor evaluations and objective measures to build a more comprehensive validity argument.

## 6 Conclusion

Notwithstanding its limitations, this study demonstrates that the CLKAQ is a reliable and valid instrument for its intended purpose. It can be effectively applied to preliminarily investigate clinical medical undergraduates’ self-perceived knowledge and attitudes toward laboratory medicine. The insights gathered can inform the development of targeted educational interventions, provide a basis for gathering structured feedback for curriculum improvement, and ultimately help refine pedagogical strategies to better meet the evolving demands of modern medical education. By serving as a catalyst for self-reflection, the CLKAQ contributes to the important goal of fostering self-regulated, lifelong learning among future physicians.

## Data Availability

The original contributions presented in this study are included in this article/Supplementary material, further inquiries can be directed to the corresponding author.

## Supplementary material

Supplementary Material 1 English version of the laboratory medicine knowledge and attitudes questionnaire.

Supplementary Material 2 Content validity assessment of the questionnaire.

Supplementary Material 3 Summary table of the coded results from 303 valid questionnaires, analyzed using SPSS 25.0 statistical software.
