# Supplementary material for: Development and validation of a questionnaire for laboratory medicine knowledge and attitudes in clinical medical interns
Source: Front Med (Lausanne). 2025 Dec 16;12:1726666. doi: 10.3389/fmed.2025.1726666 (PMC12747941; doi:10.3389/fmed.2025.1726666)
Supplement: Supplementary Material 2 — Content validity assessment of the questionnaire. [file Data_Sheet_2.docx]

**Questionnaire on Laboratory Medicine Knowledge and Attitudes Among Undergraduate Clinical Medical Interns**

**Ethics Approval**
The Ethics Committee of Hangzhou Third People's Hospital approved this study (Approval No. 2025KA064) in compliance with China's Ethical Review Measures for Biomedical Research Involving Humans and the Declaration of Helsinki.

**Research Objective**
To develop and validate a questionnaire assessing laboratory medicine knowledge and attitudes among clinical medical undergraduates during internships.

**Data Handling**

- Fully anonymized responses with no personally identifiable information.
- No IP addresses or traceable metadata collected.
- Demographic data aggregated to prevent identification.
- Data stored on encrypted servers, accessible only to the research team.

**Your Rights**

- Voluntary participation with no penalty for withdrawal.
- To withdraw consent, contact: [43754962@qq.com](mailto:43754962@qq.com).

**Consent Statement**

🗆By submitting this survey:

1. I confirm that I have read and understood the purpose and procedures.
2. I participate voluntarily and may withdraw at any time without explanation.
3. I consent to the use of my anonymized responses for research.
4. I confirm all answers reflect my genuine experiences.

**Part I Basic Information**

Gender: 🗆Male 🗆Female

Age: 🗆20-22years 🗆23-25years

City: 🗆First-tier cities 🗆Second-tier cities 🗆Third-tier cities
Internship Hospital:

**Part II Knowledge**

1. I believe my clinical laboratory medicine knowledge (e.g., report interpretation, correlation between test results and clinical diagnosis) meets the job requirements of my current institution.

🗆Strongly Disagree 🗆Disagree 🗆Neutral 🗆Agree 🗆Strongly Agree

1. I am fully aware of the clinical laboratory medicine knowledge required for my target position.

🗆Strongly Disagree 🗆Disagree 🗆Neutral 🗆Agree 🗆Strongly Agree

1. I can independently perform preliminary interpretation of common laboratory reports (e.g., Complete Blood Count (CBC), biochemistry, coagulation function).

🗆Strongly Disagree 🗆Disagree 🗆Neutral 🗆Agree 🗆Strongly Agree

1. I understand and can implement the critical value reporting system in clinical laboratory medicine.

🗆Strongly Disagree 🗆Disagree 🗆Neutral 🗆Agree 🗆Strongly Agree

**Part III Attitudes**

1. I consider clinical laboratory medicine knowledge crucial for clinical decision-making.

🗆Strongly Disagree 🗆Disagree 🗆Neutral 🗆Agree 🗆Strongly Agree

1. Acquiring more laboratory medicine knowledge provides a competitive advantage in job-seeking.

🗆Strongly Disagree 🗆Disagree 🗆Neutral 🗆Agree 🗆Strongly Agree

1. I hope to gain more clinical laboratory medicine knowledge.

🗆Strongly Disagree 🗆Disagree 🗆Neutral 🗆Agree 🗆Strongly Agree

1. What types of clinical laboratory medicine knowledge do you most want to learn? (Multiple-choice options)

🗆Interpretation of routine laboratory tests (e.g., CBC, Basic Metabolic Panel)

🗆Interpretation of test results in the context of clinical diagnosis

🗆Technical principles and operational procedures of laboratory assays

🗆Quality control and error analysis in diagnostic testing

🗆Specialized laboratory disciplines (e.g., immunology, microbiology)

1. What are your primary motivations for learning clinical laboratory medicine? (Multiple-choice options)

🗆**Enhancing Clinical Decision-Making Skills**

🗆Certification/licensing examinations

🗆Professional development requirements

🗆Intrinsic motivation for skill mastery

1. How do you typically access clinical laboratory medicine knowledge? (Multiple-choice options)

🗆Medical textbooks/literature

🗆Hospital-based training

🗆Academic lectures

🗆Online courses

🗆Mentorship

🗆Internet resources

11. What do you perceive as the main barriers to learning clinical laboratory medicine? (Multiple-choice options)

🗆Curriculum-clinical practice disconnect

🗆Time constraints

🗆Insufficient learning resources

🗆Inadequate expert mentorship

🗆Excessive content complexity

**Part IV Suggestions**

1. Do you think hospitals or medical schools should increase education on clinical laboratory medicine?

🗆Yes 🗆No

1. What suggestions do you have for improving clinical laboratory medicine training in hospitals or medical schools? (Open-ended; e.g., "Monthly online training," "Quarterly case discussions")
